# Redox mediators modify end product distribution in biomass fermentations by mixed ruminal microbes in vitro

**DOI:** 10.1186/s13568-015-0130-7

**Published:** 2015-08-04

**Authors:** Michael A Nerdahl, Paul J Weimer

**Affiliations:** Department of Bacteriology, University of Wisconsin-Madison, Madison, WI 53706 USA; United States Department of Agriculture, Agricultural Research Service, US Dairy Forage Research Center, 1925 Linden Drive West, Madison, WI 53706 USA; Department of Plant Pathology and Microbiology, Texas A&M University, 435 Nagle Street, College Station, TX 77843-2132 USA

**Keywords:** Electron mediator, Methane, Redox dye, Rumen, Volatile fatty acids

## Abstract

The fermentation system of mixed ruminal bacteria is capable of generating large amounts of short-chain volatile fatty acids (VFA) via the carboxylate platform in vitro. These VFAs are subject to elongation to larger, more energy-dense products through reverse β-oxidation, and the resulting products are useful as precursors for liquid fuels production. This study examined the effect of several redox mediators (neutral red, methyl viologen, safranin O, tannic acid) as alternative electron carriers for mixed ruminal bacteria during the fermentation of biomass (ground switchgrass not subjected to other pretreatments) and their potential to enhance elongation of end-products to medium-chain VFAs with no additional run-time. Neutral red (1 mM) in particular facilitated chain elongation, increasing average VFA chain length from 2.42 to 2.97 carbon atoms per molecule, while simultaneously inhibiting methane accumulation by over half yet maintaining total C in end products. The ability of redox dyes to act as alternative electron carriers suggests that ruminal fermentation is inherently manipulable toward retaining a higher fraction of substrate energy in the form of VFA.

## Introduction

One of the greatest needs in developing sustainable alternative energy systems is an economical means of producing energy-dense, infrastructure-compatible liquid fuels (Granda et al. 2007). Mixed cultures of microorganisms can degrade biomass to mixtures of volatile fatty acids (the carboxylate platform; Agler et al. 2011) that can then be converted by further chemistry to useful bioproducts, including liquid fuels (Holtzapple and Granda 2009; Lange et al. 2010; Levy et al. 1981). The carboxylate platform has been highlighted for its ability to be operated non-aseptically, generate high yields, and utilize a wide range of feedstocks, owing to the metabolic diversity of the undefined microbial community (Agler et al. 2011). Within the carboxylate platform, efforts have been made to improve its economics by increasing the value of products while still working within reasonable operating costs and run-times (Agler et al. 2012).

Ruminal fermentation is the means by which ruminant animals convert plant biomass to volatile fatty acids (VFA) that serve as energy source for the host animal. When conducted outside the animal (i.e., in bioreactors), the ruminal fermentation can be considered as a type of consolidated bioprocessing system that has the potential to improve upon the existing carboxylate platform for fuels and chemical production. In a manner similar to other undefined mixed cultures, such as sewage sludge or aquatic sediments, the mixed ruminal bacteria are capable of digesting a wide range of substrates (Weimer 2011). Of particular note for the ruminal bacteria is their capability to produce large amounts of short-chain volatile fatty acids (VFA) from cellulosic substrates in run times as short as 1–3 days. Likewise, the accumulation of methane in these systems is considerably lower than in most anaerobic digestion processes due to a lack of aceticlastic methanogens and proton-reducing acetogens, which require more time for growth than the short 1–3 day ruminal retention times allow, limiting their effect (Weimer et al. 2009). The ability of ruminal bacteria to produce substantial VFA yields has been well-documented and efforts have been made to elongate these short-chain VFAs to medium-chain VFAs, which are more energy-dense and more easily extractable (Singhania et al. 2013). The propensity of the ruminal bacteria towards VFA generation at considerable yield in such short run times deems it worthy of continued study with respect to chain elongation, and what low-cost methods can be used to manipulate the ruminal end-products to more valuable alternatives without altering the initial microbial composition, i.e. without addition of other bacteria. End product manipulation may also be beneficial within the rumen itself, as part of strategy for decreasing methane emissions and retaining feed energy in VFA in vivo, as this is a recognized as a fundamental goal of economically and environmentally sustainable animal agriculture (Hristov et al. 2013).

One important means of contributing to these goals is to better characterize the inherent manipulability of the ruminal fermentation under in vitro (“extraruminal”) conditions. The use of redox mediators has the potential to improve our understanding of how readily fermentation end product ratios may be altered in this system. This study examines the ability of several redox mediators—neutral red, methyl viologen, safranin O, and tannic acid—to decrease methanogenesis and shift ruminal fermentation end-products in vitro in short (72 h) run-times, as a means of demonstrating the inherent manipulability of the ruminal fermentation system.

## Materials and Methods

### Feedstocks and chemicals

Switchgrass (*Panicum virgatum* L.) air-dried whole herbage (late maturity, low quality, harvested after overwintering in February 2013) was generously provided by K. J. Shinners, University of Wisconsin-Madison. The material was ground through Wiley mill (1 mm) but otherwise was not subjected to additional pretreatment, and was stored at room temperature in the dark. Analysis (in triplicate) using the detergent fiber method of Goering and Van Soest (1970; without α-amylase treatment) revealed a composition [g (kg DM)^−1^] of: neutral detergent fiber, 878 ± 9; acid detergent fiber, 537 ± 1; and acid-detergent lignin, 86 ± 5. The N content, determined using a Leco TruMac (St. Joseph, MI, USA) combustion analyzer, was 5.2 ± 0.1 g (kg DM)^−1^. DM content of the ground switchgrass was 924 g (kg DM)^−1^.

The following redox mediators were used in their oxidized form: methyl viologen (MV, Acros, 98% dye content); neutral red (NR, Sigma, 95% dye content); safranin O (Aldrich, 96% dye content), resazurin (Sigma, ~85% dye content); tannic acid (TA, Aldrich).

### Ruminal inocula

Inocula were obtained from two lactating Holstein cows each fitted with a ruminal cannula (Bar-Diamond, Parma, ID, USA). The cows were fed a total mixed ration that contained corn silage, alfalfa haylage, ground corn grain, soybean meal and a vitamin and mineral mix. Ruminal contents (solids and liquids) from each cow were collected manually, then processed and the separate diluted ruminal fluid from each cow combined as described previously (Mouriño et al. 2001).

### Fermentations

All fermentations were conducted in triplicate within treatment, under a CO_2_ gas phase in volume-calibrated glass serum vials (Wheaton) of ~60 mL volume fitted with butyl rubber closures and aluminum crimp seals. Experiments were conducted using freshly collected and diluted ruminal inocula. Vials contained Goering–Van Soest medium (1970) reduced with cysteine and Na_2_S, along with the switchgrass [19 mg DM (mL liquid volume)^−1^]. Total liquid volume in the vials was typically 10 mL, except for the NR concentration experiment (22 mL). Unless otherwise indicated, resazurin was added at low concentrations (0.008 mM) as a redox indicator to confirm (via decolorization upon reduction) establishment of reducing conditions in the culture media. Each redox mediator was dissolved in N_2_-gassed, deionized water to achieve a ~20 mM stock solution and added to fermentation vials to achieve the indicated concentration in the medium. Each experiment included control vials that lacked redox mediators, as well as blank vials that contained media and inoculum but lacked switchgrass or redox mediators. All experimental setup and incubations were conducted under non-aseptic conditions, with no sterilization of vessels, apparatus, biomass feedstocks, or culture media. Incubations were performed 39°C in a static upright position for 72 h.

### Analysis of residual substrate and fermentation products

Analysis of gas phase H_2_ and methane was conducted by removal of fixed volumes (0.20–0.40 mL) of headspace using a pressure-lock syringe, and direct injection into a Shimadzu 8A gas chromatograph fitted with a 1.88 m × 3.18 mm (i.d.) stainless steel column packed with Carbosieve S-II (Sigma-Aldrich, St. Louis, MO, USA). The following chromatographic conditions were used: carrier gas, He; injector T, 120°C; oven T, 70°C; detector T, 120°C; detector type, thermal conductivity; detector current, 120 mA. External standard curves were used for quantification of H_2_ and CH_4_.

Culture pH was measured immediately after removal of the rubber stopper (to minimize alkalinization of medium that results from CO_2_ outgassing), using a Mettler-Toledo FiveEasy Plus pH meter calibrated with pH 4.01 and 7.00 buffers. Volatile fatty acids, nonvolatile acids (lactate, succinate) and ethanol in the culture liquid phase were determined by HPLC, as described previously (Weimer et al. 1991). For all gaseous and nongaseous products, net product formation was calculated after subtraction of products contained in substrate-free blank vials inoculated and incubated with the blank vials. Total enthalpy of combustion of products was calculated from enthalpies of combustion of individual end products (Weast 1969) at their measured net molar concentrations.

Total substrate consumption was calculated as initial dry weight of substrate minus neutral detergent fiber (NDF) residue (equivalent to plant cell wall residue). Residual NDF was determined gravimetrically by a modified Goering and Van Soest method (Weimer et al. 1990).

### Statistical analysis

Statistical tests were performed using PROC MIXED in SAS, v.9.4 (SAS, Cary, NC, USA), using the model Y_i_ = μ + S_i_ + ε_i_, where Y_i_ = dependent variable; μ = overall mean; S_i_ = effect of redox dye or its concentration; and ε_i_ = residual error. For analysis of data from the experiment conducted at different resazurin concentrations, the model Y_i_ = μ + S_i_ + R_i_ +SR_ij_ + ε_I_ was used, where R_i_ = resazurin concentration. For fermentations conducted at different neutral red concentrations, PROC REG was used for linear and quadratic regression analysis. Data are reported as least-square means. Means separation tests were conducted using the Tukey procedure. Significance was declared at *P* < 0.05, and trends identified at 0.05 < *P* < 0.10.

## Results

**Fig. 1:**
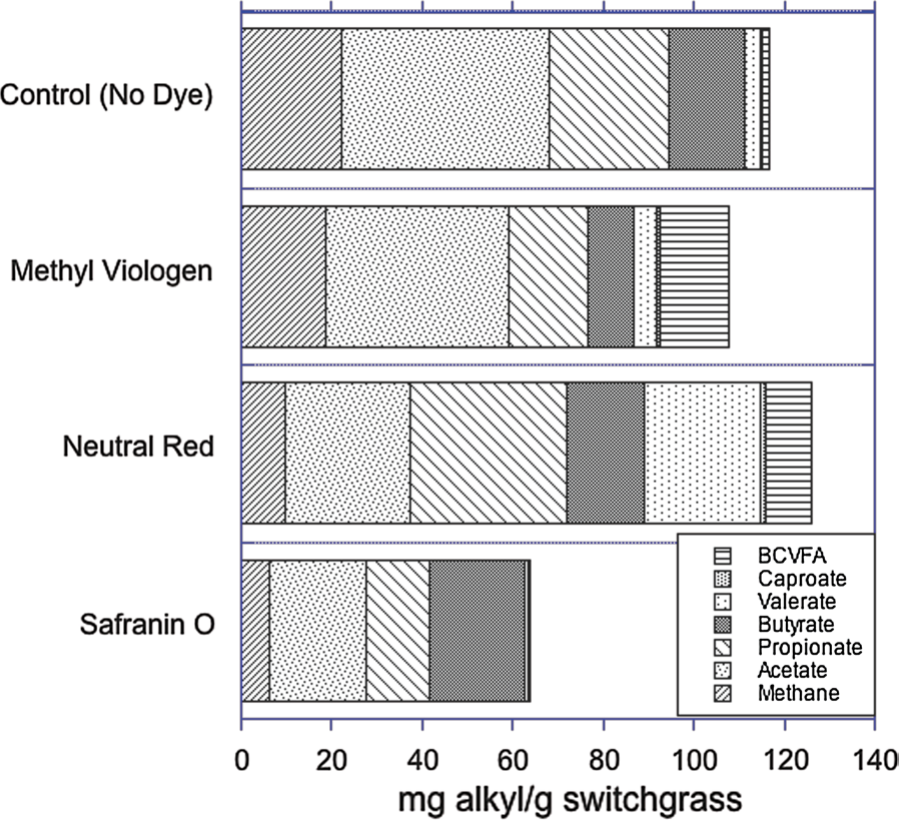
Effect of the redox dyes methyl viologen (0.5 mM), neutral red (1 mM) and safranin O (1 mM) on fermentation end product distribution by mixed ruminal microbes in vitro. Data are calculated based on the mass of alkyl groups (methyl plus methylene groups) in the indicated products, per mg of added switchgrass substrate.

Fermentations of switchgrass by the mixed ruminal inoculum yielded varying amounts of methane and C_2_–C_6_ VFA. Product concentrations were notably altered by the addition of all three redox dyes MV, NR, or safranin O (Table 1). MV addition resulted in slightly increased concentrations of caproate (C_6_) and a ninefold increase in the sum of the molar amounts of C_4_ and C_5_ branched-chain VFA (isobutyrate, isovalerate and 2-methylbutyrate), but decreased concentrations of methane and C_2_–C_4_ VFA (acetate, propionate, and butyrate), and also decreased total carbon in VFA. Safranin O largely inhibited total VFA carbon and numerically decreased caproate and valerate to near their detection limits. Butyrate production did increase, however, which led to an increase in average carbon chain length to 2.61, compared to 2.42 in the mediator-free control. NR inhibited methane production (by 56%) and significantly increased valerate, caproate, and C_4_–C_5_ branched chain VFA, and numerically increased total VFA carbon production. Comparison of the data on a per substrate added basis revealed that only NR numerically increased total end product C yield while enabling a shift in fermentation to yield longer-chain products (Fig. 1).

**Table 1:** End products from in vitro switchgrass fermentations by mixed ruminal microorganisms in the presence or absence of redox dyes

Product	Net umol product^a^				SED^b^	Model *P* > F
	Methyl viologen (0.5 mM)	Neutral red (1.0 mM)	Safranin O (1.0 mM)	Control (no dye)		
Methane	122.1 A	62.1 B	38.5 B	139.2 A	9.9	<0.0001
Acetate	269.8 A	182.0 B	142.9 B	305.6 A	13.7	<0.0001
Propionate	60.4 C	119.7 A	48.0 C	91.0 B	5.0	<0.0001
Butyrate	24.0 B	40.2 AB	48.8 A	38.8 AB	5.9	0.018
Isobutyrate	9.7 A	4.3 B	0.8 C	1.8 C	0.7	<0.0001
Valerate	6.8 B	35.9 A	1.5 C	5.3 BC	1.4	<0.0001
Isoval + 2 MB^c^	19.1 A	14.4 B	0.1 C	1.4 C	1.0	<0.0001
Caproate	6.7 A	8.1 A	0.3 B	2.1 B	1.6	0.003
Total VFA	399.1 A	404.1 A	242.3 B	441.5 A	15.4	<0.0001
Total VFA-C	1025.6 B	1201.3 A	629.6 C	1,092.0 AB	40.5	<0.0001
ACL^d^	2.58 B	2.97 A	2.61 B	2.42 C	0.04	<0.0001
Enthalpy of combustion, kJ	0.602 B	0.667 A	0.343 C	0.633 AB	0.009	<0.0001

Vials contained 100 mg switchgrass and 3.0 mL of diluted mixed ruminal inoculum in a total liquid volume of 10 mL.

^a^Values are least-square means from triplicate cultures after 72 h incubation, after correction for inoculated blank vials lacking switchgrass. Values within row having different letters (A, B, C) differ (*P* < 0.05).

^b^Pooled standard error of the difference.

^c^Isovalerate plus 2-methylbutyrate (chromatographically co-eluting isomers).

^d^Average chain length of VFA.

**Fig. 2:**
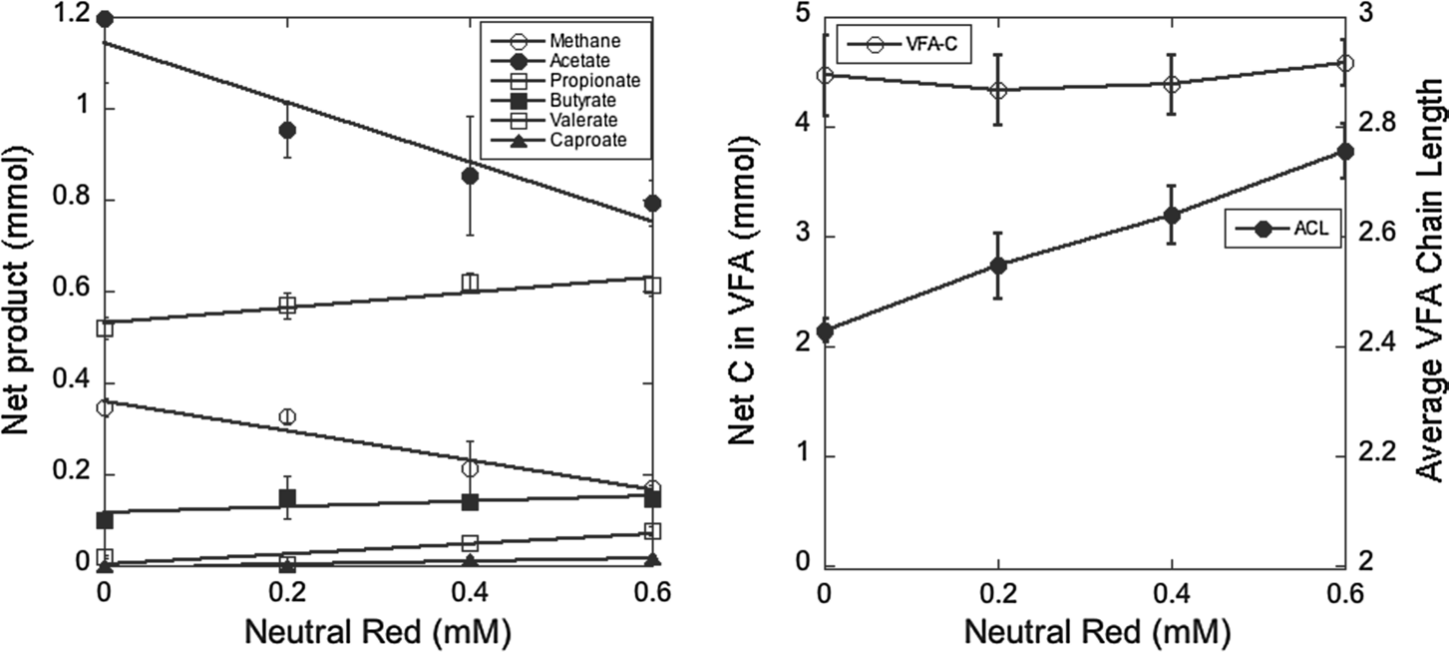
Effect of neutral red concentration of fermentation end products by mixed ruminal microbes in vitro. Results are mean values from triplicate cultures. *Error bars* indicate standard errors of the mean.

Owing to the significant improvement in C_5_ and C_6_ VFA production resulting from NR addition, the effect of NR concentration on the fermentation was examined (Fig. 2). Addition of NR in increasing concentrations up to 0.6 mM resulted in linear increases in propionate (*P* < 0.001), butyrate (*P* = 0.099), valerate (*P* = 0.002), caproate (*P* < 0.001) and average VFA chain length (ACL; P < 0.0001). Caproate concentrations increased linearly (*P* < 0.001) from an undetectable concentration (< 0.05 mM) in the absence of NR to 2.2 mM at the highest NR concentration, and valerate levels increased by over fourfold to 8.8 mM. These increases were accompanied by linear decreases (*P* < 0.0001) in both methane and acetate. At 0.6 mM NR, the highest concentration tested, methane production decreased by 52% relative to the mediator-free control. Total VFA carbon did not significantly differ among the range of NR concentrations tested (range 434–459 mM, SED = 24.4 mM, *P* = 0.59).

Another possible redox mediator, tannic acid, was tested to determine whether it could also alter fermentation product distribution (Table 2). The addition of tannic acid in concentrations of 0.1 and 0.3 g L^−1^ produced minimal effects on average chain length (ACL) and total VFA yield. The highest level of tannic acid tested, 1.0 g L^−1^, resulted in a decrease in the amount of switchgrass digested; a slight suppression methane and propionate yields; and a slight decrease in overall ACL of the VFA.

**Table 2:** Effect of tannic acid (TA) on in vitro switchgrass (SWG) fermentations by mixed ruminal microorganisms

Variable	Least-square mean values^a^				SED^b^	Model *P* > F
	Control (no TA)	0.1 g TA L^−1^	0.3 g TA L^−1^	1.0 g TA L^−1^		
SWG digested (mg DM)	79.3 A	79.8 A	76.9 A	70.0 B	2.2	0.007
mmol product (g SWG consumed)^−1^						
Methane	1.29 A	1.40 A	1.19 AB	1.02 B	0.079	0.015
Acetate	5.14	5.42	5.17	5.31	0.906	0.906
Propionate	2.10 A	2.22 A	1.95 AB	1.58 B	0.154	0.015
Butyrate	0.342	0.420	0.345	0.334	0.052	0.370
Valerate	0.008	0.011	0.032	0	0.015	0.246
Caproate	0.013	0.018	0.014	0.014	0.003	0.197
BCVFA^c^	0.022	0.050	0.014	0.002	0.017	0.104
Total VFA	7.62	8.13	7.52	7.13	0.68	0.568
Total VFA-C	18.17	19.59	17.89	16.78	1.317	0.430
Total alkyl in SCFA^d^	10.54	11.40	10.33	9.12	1.057	0.267
ACL^e^	2.38 A	2.40 A	2.37 A	2.27 B	0.024	0.024
Final pH	6.41	6.41	6.39	6.42	0.051	0.940
Enthalpy of combustion, kJ (g SWG digested)^−1^	9.74	10.51	9.52	8.77	0.827	0.287

Vials contained 190 mg switchgrass DM and 2.0 mL of diluted mixed ruminal inoculum in a total liquid volume of 10 mL.

^a^Values are from triplicate cultures after 72 h incubation, after correction for inoculated blank vials lacking SWG. Values within row having different letters (A, B) differ (*P* < 0.05).

^b^Pooled standard error of the difference.

^c^Branch-chain C_4_–C_5_ VFA (sum of isobutyrate, 2-methylbutyrate, and isovalerate).

^d^Sum of methyl and methylene groups in VFA.

^e^Average chain length of VFA.

In order to test whether the redox indicator resazurin contributed to the shift in fermentation products in the above experiments (i.e., by acting as a primary or secondary electron carrier), fermentations were conducted in which resazurin was added at concentrations of 0, 0.008, 0.03, 0.10, 0.25, and 0.45 mM. No differences were observed among these treatments with respect to substrate consumption or product formation (Table 3). In addition, switchgrass fermentations were conducted with 0.5 mM NR in the presence or absence of 0.008 mM resazurin (the concentration used in other experiments). As in previous experiments, the addition of NR resulted in significantly lower production of methane as well as acetate with significantly higher production of valerate (although, unexpectedly, no caproate) and increased average VFA chain length. However, the addition of resazurin (0.008 mM) with NR produced no additional increases or decreases in end product concentrations. Thus it does not appear that resazurin can serve as an electron carrier in these fermentations.

**Table 3:** Effect of resazurin (Res) and Neutral Red (NR) on fermentation of switchgrass (SWG) by mixed ruminal microflora in vitro

Variable	Resazurin (Res, in mM) without Neutral Red (NR)						0.5 mM NR		SED^a^	*P* > F, Res^b^	P > F, NR^c^
	No Res	Res (0.008)	Res (0.03)	Res (0.10)	Res (0.25)	Res (0.45)	No Res	Res (0.008)			
SWG digested(mg)	74.9 A	75.2 A	73.3 AB	72.2 AB	73.3 AB	70.9 AB	66.7 ABC	64.3 BC	2.39	0.597	0.967
Net μmol											
H_2_	0.13 B	0.14 B	0.10 B	0.25 B	0.27 B	0.09 B	2.23 A	2.42 B	0.29	0.154	0.999
Methane	110.0 A	109.6 A	108.4 A	107.3 A	107.7 A	106.1 A	37.6 B	35.2 B	3.72	0.932	0.997
Formate	0 B	1.3 B	0 B	0 B	0 B	0 B	23.7 A	18.1 A	4.0	0.785	0.846
Acetate	359.1 A	339.0 A	338.2 A	336.7 A	337.4 A	300.0 A	222.7 B	208.5 B	18.3	0.176	0.990
Propionate	140.1	148.2	137.5	143.8	144.7	138.4	160.5	153.7	7.44	0.714	0.976
Butyrate	30.4	26.2	27.7	25.9	26.9	25.7	30.4	27.9	3.50	0.573	0.995
Isobutyrate	1.3 AB	2.1 A	1.7 A	0.9 AB	1.5 AB	1.0 AB	0	0	1.43	0.314	1.000
Valerate	4.0 AB	2.0B	2.5 B	2.2 B	2.2 B	2.2 B	5.6 A	5.5 A	0.78	0.227	1.000
Isovalerate + 2 MB	4.7	2.6	4.4	2.2	3.3	2.4	0	0	1.4	0.585	1.000
Caproate	0.31	0.38	0	0	0	0	0	0	0.14	0.113	1.000
Total C_2_–C_6_ VFA	534.1 A	520.5 A	512.1 A	511.6 A	516.0 A	469.9 AB	416.7 B	391.3 B	25.6	0.277	0.953
Total VFA alkyl^d^	752.0 A	741.0 A	729.8 A	722.1 A	733.7 A	675.6 AB	647.3 B	604.5 B	33.0	0.313	0.867
Total alkyl^e^	861.8 AB	850.6 AB	838.2 AB	829.4 AB	841.5 AB	781.7 AB	684.9 BC	639.7 C	34.1	0.294	0.854
ACL^f^	2.41 B	2.42 B	2.43 B	2.41 A	2.42 A	2.44 B	2.55 A	2.55 A	0.024	0.454	0.997
μmol H (mg SWG)^−1^	41.31	39.44	39.94	40.14	39.97	38.30	34.48	35.34	1.88	0.806	0.965
Combustion, kJ (g SWG digested)^−1^	9.94	9.51	9.65	9.67	9.65	9.26	8.65	8.46	0.83	0.832	0.608

Values are least-square means from triplicate cultures that contained 190 mg switchgrass (DM basis), 2.0 mL diluted ruminal inoculum and 10 mL total liquid volume. End product amounts from 72 h fermentations have been corrected for inoculated blank vials that lacked SWG.

^a^Pooled standard error of the difference.

^b^Comparison of least-square means across all treatments not containing NR (0–0.45 mM resazurin).

^c^Comparison of least-square means between NR and NR + 0.008 mM resazurin.

^d^Sum of methyl plus methylene groups in C_2_–C_6_ VFA.

^e^Includes methane.

^f^Average chain length of VFA (excluding formate).

*ABC* different letters within row indicate differences in least-square means (*P* < 0.05).

In the above fermentations inhibition of methanogenesis by NR was accompanied by accumulation of modest amounts of both H_2_ and formate. Fermentations containing either 0 or 0.008 mM resazurin with NR (Table 2) averaged 34.9 μmol H atoms in measured fermentation products (mg switchgrass digested)^−1^, numerically less than the 40.5 μmol H atoms (mg switchgrass digested)^−1^ in parallel fermentations lacking NR, but this difference was not significant. However, total H atoms in measured fermentation products (mg switchgrass digested)^−1^ tended (*P* = 0.089) to be lower in the presence versus absence of NR without resazurin, and was nearly so (*P* = 0.115) with 0.008 mM resazurin, suggesting that there may be additional fermentation products not accounted for in the NR-supplemented cultures. As expected, all cultures had similar enthalpies of combustion on a per mg switchgrass digested basis.

## Discussion

Shifting metabolic products of plant matter degradation to decrease methanogenesis and increase VFA is desirable within the rumen itself (as it should increase energetic efficiency and decrease greenhouse gas emissions) and in extraruminal bioreactors (as it should foster chain elongation to higher-energy, medium chain-length carboxylates). The inherent manipulability of the ruminal fermentation has largely been explored by altering substrate (i.e., diet composition) or by adding specific metabolic inhibitors (e.g., of methanogenesis), but the limits to which the fermentation can be altered, and the mechanisms underlying these alterations, have not been firmly established (Ungerfeld 2015). In this study we used artificial electron acceptors (redox mediators) to examine fermentation end product distribution on a single plant biomass substrate (ground switchgrass not subjected to chemical pretreatment).

The capability of redox dyes to shift anaerobic fermentation towards formation of longer end-products was first examined in non-ruminal systems by Hongo (1958). By diverting reducing equivalents that are normally released as H_2_ towards formation of NAD(P)H, redox dyes can inhibit methanogenesis by decreasing the supply of H_2_, and provide NAD(P)H for use as reducing power to synthesize longer chain molecules. Whether a redox mediator may divert electron flow depends on its capability to compete with a natural electron carrier in vivo. MV has a similar redox potential to ferredoxin and thus may replace ferredoxin in hydrogenase-catalyzed reactions (Peguin et al. 1994). That MV may replace ferredoxin allows it to act as a substitute for the direct reduction of NAD^+^. Accumulation of NADH inhibits further NAD^+^ reduction by either MV or ferredoxin (Rao and Mutharasan 1987). This is the mechanism for production of longer chain products with redox dyes: because a second electron carrier is present, the natural electron carrier will accumulate unless the reaction is coupled to the further synthesis of longer chain molecules that decreases the amount of NADH present in the cell.

Of the redox mediators tested, NR was most effective at decreasing methane production and facilitating VFA chain length extension. The reduction potential of NR (Table 4) is very close to that of NADH (−0.32 V). Because of its similar redox potential, neutral red may act as an electron carrier and perform in enzymatic reactions in a manner similar to NAD^+^/NADH. Because its reduction potential is higher than that of ferredoxin (approximately −0.39 V), NR could potentially accept electrons from reduced ferredoxin, decreasing H_2_ production. Although studies on H_2_ oxidation coupled to ferric iron reduction by *Escherichia coli* suggested that NR stimulated both H_2_ and formate oxidation (McKinlay and Zeikus 2004), NR addition resulted in accumulation of both H_2_ and formate in our studies with mixed ruminal microbes. As these two compounds are the primary electron donors in ruminal methanogenesis, it appears that NR’s inhibition of methanogenesis may occur via interference with other redox reactions within the methanogenic pathway itself, rather than by inhibiting fermentative production of, or stimulating oxidation of, H_2_ and formate. It would be of fundamental interest to determine if classical inhibitors of methanogenesis, such as 2-bromoethanesulfonic acid or chlorinated methane analogs, could augment the effects of NR in inhibiting methanogenesis and further extending carbon chain length.

**Table 4:** Reduction potentials of redox mediators

Redox mediator	E° (V)	References
Methyl viologen	−0.446	Michaelis and Hill (1933)
Neutral red	−0.325	Park et al. (1999)
Safranin O	−0.279	Stieliler et al. (1933)
Resazurin	+0.042	Hungate (1969)
Tannins	+0.571 to +0.996^a^	Hagerman et al. (1998)

^a^Varies with specific compound; range is at experimental pH of 6.24, similar to pH values of fermentation experiments reported here.

An increased availability of NADH leads to increased production of VFAs, such as valerate and caproate. This is accomplished by reverse β-oxidation, which sequentially adds acetyl (C_2_) units to form butyrate from two molecules of acetate, valerate from acetate and propionate, and caproate from acetate and butyrate (Agler et al. 2012). This is most clearly demonstrated by a sevenfold increase in valerate upon the addition of 1 mM NR (Table 1). Interestingly, the levels of propionate were higher in the NR cultures than in the control, while acetate levels decreased by almost half. Not only did NR increase the concentration of C_5_–C_6_ VFAs, but also C_3_–C_4_ VFAs (propionate and butyrate) as well. So while the average chain length of end-products increased from 2.42 to 2.97, decreases in end-products only occurred with acetate and methane, the least desirable products in terms of energy content and commercial value.

The levels of total carbon in VFA resulting from MV or NR additions were nearly identical. Of interest is that MV did a significantly poorer job of inhibiting methanogenesis and facilitating chain elongation of acetate. Although Bauchop (1967) demonstrated that the related dye benzyl viologen decreased methanogenesis by mixed ruminal microbes in vitro, the effects on the amounts and distribution of other end products were not reported. The capability of MV to serve as an electron carrier for hydrogenase and increase the availability of NADH for VFA chain elongation was observed by Peguin et al. (1994) who showed MV to increase butyrate concentrations to 0.65 mol (mol glucose)^−1^ in pure cultures of the non-ruminal solventogenic bacterium, *Clostridium acetobutylicum*. In our experiments, the amount of butyrate was numerically lowered by MV, but the caproate concentration rose to over three times that of the control. This confirms not only that MV is capable of generating butyrate at the expense of shorter products, but also that a second chain elongation to caproate took place despite the short run times, although to relatively modest concentrations.

Of further interest is that NR shifted the fermentation to longer-chain end-products in a concentration-dependent manner. NR was also unaffected by the addition of resazurin as a redox indicator for reducing conditions of the ruminal media. Within the range of concentrations tested, linear decreases in acetate and methane along with increases in C_3_–C_6_ VFA occurred upon additions of greater amounts of NR. This demonstrates that there may be no specific threshold for NR activity. At what NR concentration the linear functions of methane inhibition and VFA chain extension dissipate may depend on the toxicity of the end-products.

At low concentrations, TA had no effect on fermentation product profiles, probably due to its relatively positive redox potential. At the highest concentration tested (1.0 g L^−1^), TA displayed a slight inhibition of switchgrass degradation and a corresponding decrease in methane and propionate formation, and in the ACL of VFA products. In addition, it decreased yield of C_4_–C_5_ branched-chain VFA, which are considered to be produced exclusively by fermentation of branched-chain amino acids (Russell 2002). It is likely that the TA-mediated inhibition of branched-chain VFA production reflects the known ability of tannins to bind proteins and protect them from ruminal degradation (Min et al. 2003).

In this report, we show that certain redox mediators may be an effective means of extending carbon chain length, and we highlight NR as the most effective of the dyes examined. NR is capable of increasing the average carbon chain length of end-products from 2.42 to 2.97 and inhibiting methanogenesis by over half, all while including no additionally added substrates/reagents and keeping fermentation run times at 72 h. The fact that shifts in end product distribution vary dramatically among different redox mediators that differ only slightly in their reduction potential reinforces bioenergetics studies that the ruminal fermentation is primarily under thermodynamic control (Ungerfeld and Kohn 2006). The manipulability of the ruminal system to form higher value fermentation end-products in vitro is clear, and it is a system worthy of further study to identify other, more practical interventions for shifting end product distribution, for potential use on an industrial scale.
